# Sulforaphane Combined with Vitamin D Induces Cytotoxicity Mediated by Oxidative Stress, DNA Damage, Autophagy, and JNK/MAPK Pathway Modulation in Human Prostate Tumor Cells

**DOI:** 10.3390/nu15122742

**Published:** 2023-06-14

**Authors:** Katiuska Tuttis, Ana Rita Thomazela Machado, Patrick Wellington da Silva Santos, Lusânia Maria Greggi Antunes

**Affiliations:** 1 Department of Genetics, Ribeirão Preto School of Medicine, University of São Paulo—USP, Ribeirão Preto 14049-900, SP, Brazil; 2 Department of Clinical Analysis, Toxicology, and Food Sciences, Ribeirão Preto School of Pharmaceutical Sciences, University of São Paulo—USP, Ribeirão Preto 14040-903, SP, Brazil

**Keywords:** calcitriol, nutrigenomics, chemoprevention, nutraceuticals

## Abstract

Prostate cancer ranks second in incidence worldwide. To date, there are no available therapies to effectively treat advanced and metastatic prostate cancer. Sulforaphane and vitamin D alone are promising anticancer agents in vitro and in vivo, but their low bioavailability has limited their effects in clinical trials. The present study examined whether sulforaphane combined with vitamin D at clinically relevant concentrations improved the cytotoxicity of the compounds alone towards DU145 and PC-3 human prostate tumor cells. To assess the anticancer activity of this combination, we analyzed cell viability (MTT assay), oxidative stress (CM-H_2_DCFDA), autophagy (fluorescence), DNA damage (comet assay), and protein expression (Western blot). The sulforaphane–vitamin D combination (i) decreased cell viability, induced oxidative stress, DNA damage, and autophagy, upregulated BAX, CASP8, CASP3, JNK, and NRF2 expression, and downregulated BCL2 expression in DU145 cells; and (ii) decreased cell viability, increased autophagy and oxidative stress, upregulated BAX and NRF2 expression, and downregulated JNK, CASP8, and BCL2 expression in PC-3 cells. Therefore, sulforaphane and vitamin D in combination have a potential application in prostate cancer therapy, and act to modulate the JNK/MAPK signaling pathway.

## 1. Introduction

Prostate cancer is currently the second most commonly diagnosed cancer and the fifth leading cause of cancer-related deaths among men worldwide [1]. Additionally, there has been a concerning rise in prostate cancer cases among individuals aged between 15 and 40 years, with an increase of 2% annually since 1990 [2].

Surgery or radiation therapy can be used effectively in most cases of cancer in the prostate and adjacent tissues, while androgen deprivation is the standard therapy to treat advanced or metastatic cancer. Unfortunately, androgen deprivation loses its effectiveness over time; i.e., tumor cells become resistant to this therapy and proliferate even in the absence of androgens, and the disease progresses to metastatic castration-resistant prostate cancer. Even with the introduction of new therapeutic approaches, patients who undergo these treatments only experience a 4 to 6-month extension in survival compared to those who receive placebo treatment [3].

The concept of cancer chemoprevention involves the utilization of both synthetic and natural compounds to decelerate or inhibit the progression of cancer [4]. While certain natural chemopreventive compounds may possess toxicity, numerous compounds exhibit low levels of toxicity and are well-tolerated. According to pre-clinical studies, a variety of natural products and dietary agents have the potential to prevent or delay the development, progression, and recurrence of cancer [5]. Epidemiological studies have proven the significance of dietary habits in promoting health and preventing diseases [6]. In this sense, cancer chemoprevention using nutraceuticals as a viable and non-toxic option has been widely studied [7]. In addition, nutraceuticals can modulate several signaling pathways in cancer cells. The synergistic anticancer effects of the combination of these nutraceuticals represent a promising alternative to overcome possible limitations due to their low bioavailability [8].

Sulforaphane, from cruciferous vegetables such as broccoli, is a potential chemopreventive agent. It is a pleiotropic compound capable of targeting several cancer-related processes, including cell proliferation, apoptosis, angiogenesis, and metastasis [9,10]. In preclinical studies, sulforaphane (i) promoted detoxification of airborne carcinogens by activating the Nrf2 pathway and phase II enzymes; (ii) exerted anti-inflammatory effects against acute lung inflammation [11]; (iii) caused apoptosis, proliferation inhibition, and cell cycle arrest in bladder cancer cell lines [9]; and (iv) protected HUVEC (human umbilical vein endothelial cell) cells from angiotensin II-induced damage by decreasing oxidative stress and mitochondrial damage [12].

Promising results were observed in a clinical trial in which men with biochemical recurrence after radical prostatectomy were given a daily dosage of 60 mg of sulforaphane through a commercial dietary supplement for a duration of six months [13]. The anticancer properties of sulforaphane have been previously studied, but its therapeutic significance has been limited by the challenge of administering it in appropriate doses. To address this limitation, a combination of anticancer compounds that target distinct functional pathways may enhance their anticancer activity and improve their effectiveness in cancer chemoprevention [14,15,16].

The synthesis of Vitamin D3 occurs in the skin when exposed to ultraviolet B radiation, and it is also present in animal-derived foods [17]. Vitamin D plays a crucial role in maintaining the balance of calcium and phosphate for proper bone mineralization. Additionally, it regulates various signaling pathways associated with cell proliferation, apoptosis, differentiation, inflammation, invasion, angiogenesis, and metastasis, which may impact cancer growth and development [18]. Many preclinical studies have revealed that high concentrations of vitamin D exposure inhibit the growth of prostate cancer cells in vitro and even delay tumor growth in animal models [19].

Clinical studies have evidenced that individuals diagnosed with prostate cancer often have insufficient levels of vitamin D in their bloodstream, and its severe deficiency is associated with more advanced stages of the disease [20,21]. However, patients who consume high doses of vitamin D show improved values of phase angle as measured by bioimpedance, indicating better nutritional status and overall health [22]. As single agents generally have limited effects in the clinical therapy of cancer, the combination of other compounds with vitamin D has been explored to enhance their antitumor efficacy [23].

Administration of vitamin D combined with sulforaphane decreases the incidence and size of tumors in mice intestine, reduces histone deacetylase activity, and increases autophagy induction [24]. Sulforaphane and vitamin D alone are promising anticancer agents, but they have demonstrated low efficacy and bioavailability in clinical trials. Hence, it is necessary to develop new strategies to enhance their effects at clinically relevant doses. The present study hypothesizes that vitamin D combined with sulforaphane potentiates their anticancer effects through induction of cytotoxicity, oxidative stress, autophagy, and DNA damage in DU145 and PC-3 metastatic human prostate tumor cells.

## 2. Materials and Methods

### 2.1. Chemical Agents and Reagents

Sulforaphane (CAS 4478-93-7), calcitriol (vitamin D in active form, CAS 32222-06-3), trypan blue (CAS 72-57-1), dimethyl sulfoxide (DMSO, CAS 67-68-5), Triton X-100 (CAS 9036-19-5), methyl methanesulfonate (MMS, CAS 66-27-3), hydrogen peroxide (CAS 7722-84-1), and ethanol (EtOH, CAS 64-17-5) were obtained from Sigma-Aldrich (Saint Louis, MO, USA). 3-[4,5-Dimethyl-thiazol-2-yl]-2,5-diphenyltetrazolium bromide (MTT, CAS 298-93-1) was obtained from Invitrogen (Waltham, MA, USA). RPMI 1640 culture medium, DMEM (Dulbecco’s Modified Eagle Medium), antibiotic mix (penicillin/streptomycin/neomycin), fetal bovine serum, and TrypLE™ Express were acquired from Gibco (Grand Island, NY, USA).

### 2.2. Cell Culture Conditions

The cell lines utilized in this study were obtained from the American Type Culture Collection (ATCC). PC-3 cells (metastatic prostate adenocarcinoma cells in bone—CRL-1435) were cultivated in RPMI 1640, while DU145 cells (metastatic prostate adenocarcinoma cells in the brain—HTB-81) were grown in DMEM medium. Both culture media were supplemented with 10% fetal bovine serum and 1% antibiotic mix. The cell culture procedures were conducted in a Class II and type A1 laminar flow hood (VecoFlow Ltda.—Campinas, SP, Brazil), following the cell line maintenance protocols proposed by Bal-Price and Coecke [25]. In all assays, solvent (0.2% DMSO + 0.2% EtOH) and positive control groups (150 µM MMS, 100 µM chloroquine, or 1 mM H_2_O_2_) were included, according to each protocol reported in the literature. The cultures were maintained at 37 °C in an atmosphere containing 5% CO_2_ and 95% relative humidity. Cells between the third and eighth passage after thawing were used for the experiments.

### 2.3. Selection of Sulforaphane and Vitamin D Concentrations

To study the in vitro biological activity of dietary bioactive compounds, it is important to select concentrations corresponding to the highest physiological concentration found in plasma, as their bioavailability is a limiting factor for administration to humans [15,26]. The concentrations used for the assays were selected from literature data that considered the maximum plasma concentration found and the maximum tolerated dose. Thus, the sulforaphane concentrations selected were 2, 4, and 8 µM, due to the sulforaphane plasma concentration of 7.3 µM detected after ingestion of broccoli with a high content of glucosinolates [27]. The vitamin D concentration selected was 16 nM, in agreement with the plasma concentration of calcitriol in patients treated with the maximum tolerated dose, 74 µg/week, intravenously [28].

### 2.4. Cell Viability Assay

The MTT assay was conducted as proposed by Mosmann [29]. Cells (1 × 10^4^/well) were seeded in 96-well culture plates, incubated for 24 h, and treated with sulforaphane and/or vitamin D for a further 24 h. The MTT solution (0.5 mg/mL) was added to each well for 3 h at 37 °C. Next, the formazan crystals were dissolved with 200 µL DMSO. Absorbance was recorded in a spectrophotometer (Biotek ELX800, Winooski, VT, USA) set at 570 nm. The presented results indicate the percentage of viable cells in relation to the solvent control.

### 2.5. Oxidative Stress Assay

To assess the oxidizing activity, we used the marker CM-H_2_DCFDA (Life Technologies, Eugene, OR, USA), following the manufacturer’s recommendations. DU145 and PC-3 cells (1 × 10^4^/well) were seeded in black 96-well plates with a clear bottom, stabilized for 24 h, and treated with sulforaphane and/or vitamin D for 3 h. After exposing the cultures to CM-H_2_DCFDA, H_2_O_2_ was added to the positive control wells for 20 min. Fluorescence was recorded in a Synergy 2 spectrophotometer (BioTek; Winooski, VT, USA) using excitation and emission filters set at 450 and 520 nm, respectively. The presented results show the percentage of reactive species in relation to the solvent control, with the fluorescence values of the solvent control being considered 100%.

### 2.6. Autophagy Assay

The detection of autophagic vacuoles was performed following the method described by Machado et al. [30], as well as the specifications provided by the manufacturer of the Autophagy Assay Kit (ab139484, Abcam, Cambridge, UK). PC-3 and DU145 cells (1 × 10^4^) were seeded in a 96-well plate, stabilized for 24 h, and treated for further 24 h with sulforaphane and/or vitamin D. Fluorescence was recorded in a Synergy 2 spectrophotometer (BioTek^®^; Winooski, VT, USA) using Excitation/Emission filters of 463/534 nm and 350/461 nm. The results are presented as the autophagy ratio proposed by Tusskorn et al. [31]. The images were acquired with the Leica DMI 6000B Fluorescence Microscope (Leica; Wetzlar, HE, Germany) from Confocal Microscopy Multiuser Laboratory (LMMC).

### 2.7. Comet Assay

DNA damage was analyzed using the comet assay, following the protocol reported by Tice et al. [32]. Cells (5 × 10^4^/well) were seeded in 24-well plates, stabilized for 24 h and treated with sulforaphane and/or vitamin D for further 24 h. The automatic cell counter Countess (Thermo Fisher, Waltham, MA, USA) was used to determine the cell viability of the samples through the Trypan Blue exclusion method. All the viabilities were greater than 70%. To prepare the slides, 150 μL of 0.5% low melting point agarose were added to the samples. A 60 μL aliquot of the suspension of each sample was applied to the slides previously covered with 1.5% normal agarose. Slides were kept in lysis solution (2.5 M NaCl, 100 mM EDTA, 10 mM Tris, 1% Triton X-100, and 10% DMSO, pH 13) overnight at 4 °C, washed, and stored in an electrophoresis tank with alkaline solution (300 mM NaOH and 1 mM EDTA, pH > 13) for 20 min in an ice bath. Electrophoresis was performed in an alkaline solution for 20 min under constant voltage (0.87 V/cm) and amperage (300 mA). After electrophoresis, the slides were immersed in neutralization solution (0.4 M Tris, pH 7.5) for 5 min, dried at room temperature, and fixed in absolute ethanol for 5 min.

The slides were stained with GelRed™ 1:10,000 (*v*/*v*) (Uniscience, São Paulo, SP, Brazil) for 3 min, and analyzed under an AxioStar Plus fluorescence microscope (Zeiss) equipped with a camera, using a 515–560 nm filter, 590 nm barrier filter, and 20× objective. Three hundred nucleoids were analyzed for each treatment, using Comet Assay IV™ software (Instem plc, Stone, ST, UK). The results are presented as DNA percentage in the comet’s tail (tail intensity).

### 2.8. Protein Extraction and Western Blot Assay

The protein extraction and Western blot assay was performed according to Machado et al. [30]. Cell lines were seeded in 75 cm^2^ ventilated cell culture flasks (2 × 10^6^ cells per flask) and treated with sulforaphane and/or vitamin D for 24 h. Next, proteins were extracted and quantified using the Pierce™ BCA Protein Assay kit (Thermo Fisher Scientific. Waltham, MA, USA).

Protein separation was achieved using SDS-polyacrylamide gel electrophoresis, followed by a transfer onto a nitrocellulose membrane. Subsequently, primary antibodies were applied overnight at 4 °C, and HRP-conjugated secondary antibodies were applied for 1 h (Table 1). The ChemiDoc™ system (Bio-Rad, Hercules, CA, USA) was utilized for visualizing protein bands, and their quantifications were performed using ImageJ 1.53 software (National Institutes of Health, Bethesda, MD, USA).

### 2.9. Prediction of Interactions

The sulforaphane and vitamin D interactions with the analyzed proteins were predicted using the STITCH v5.0 database. STITCH incorporates data on interactions within metabolic pathways, crystal structures, binding experiments, and relationships between targets and compounds. The integration of information derived from phenotypic effects, text mining, and chemical structure similarity enables the prediction of relationships between chemical substances [33]. To analyze the interactions, eight terms were added to the search (sulforaphane, calcitriol, MAPK8, CASP3, CASP8, BCL2, BAX, and NFE2L2), using Homo sapiens as the selected organism.

### 2.10. Statistical Analysis

To determine the normal distribution of the data, the Shapiro–Wilk normality test was conducted. For the treatments involving sulforaphane or vitamin D alone, data were analyzed using a one-way analysis of variance (ANOVA), followed by Tukey’s test. The combined treatment data were analyzed using the highest single agent approach. The P value from comparison between the combined and isolated treatments was the parameter used to detect a positive effect. A two-way ANOVA, followed by Tukey’s test, was employed to compare the three treatments (sulforaphane alone, vitamin D alone, and their combination). The combination index (CI) was calculated using the following formula:

(1) CI=Es, Ev/Esv

where Es = sulforaphane effect, Ev = vitamin D effect, Esv = combined treatment effect. CI < 1 indicates a positive combination effect, and CI > 1 indicates a negative combination effect [34].

The positive control groups and protein expression levels were analyzed using Student’s *t*-test. Three independent experiments were performed for each assay. The means were considered significantly different when *p* < 0.05. All statistical analyses were conducted using GraphPad Prism 8 software, version 8.0.1 (GraphPad Software, San Diego, CA, USA).

## 3. Results

### 3.1. Analysis of Cell Viability

**Figure 1:**
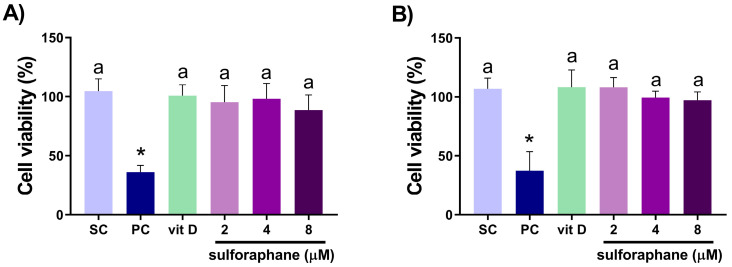
Percentage of cell viability of DU145 (**A**) and PC-3 (**B**) treated with vitamin D (16 nM) or sulforaphane (2, 4, and 8 µM) for 24 h, evaluated via the MTT assay. SC = solvent control (0.2% EtOH + 0.2% DMSO); PC = positive control (150 µM MMS). Mean ± standard deviation (*n* = 3). Values that do not differ from each other share the same letter (one-way ANOVA followed by Tukey’s test, *p* < 0.05). * Different from the solvent control (Student’s *t*-test, *p* < 0.05).

**Figure 2:**
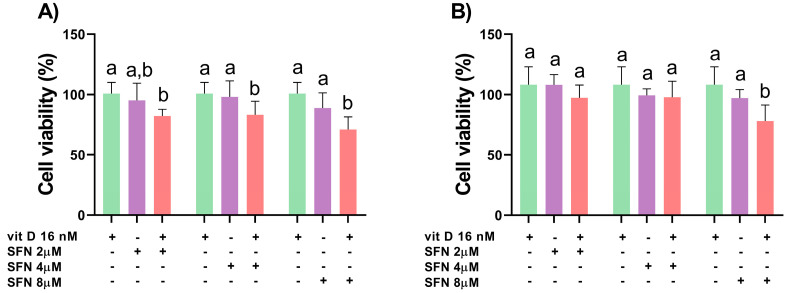
Percentage of cell viability of DU145 (**A**) and PC-3 (**B**) treated with sulforaphane (SFN; 2, 4, and 8 µM) and vitamin D (VitD; 16 nM) for 24 h, evaluated via the MTT assay. Data are expressed as mean ± standard deviation of three independent experiments. Distinct letters represent significant intra-group differences (two-way ANOVA followed by Tukey’s test, *p* < 0.05).

Vitamin D (16 nM) and sulforaphane (2, 4, and 8 µM) alone did not decrease cell viability in both cell lines (Figure 1). Compared with the isolated compounds, the 24 h treatment with vitamin D and sulforaphane at 4 and 8 µM in combination reduced the cell viability of DU145 cells, with CI = 0.12 and CI = 0.41, respectively. The effect of sulforaphane at 2 µM combined with vitamin D was equal to treatment with sulforaphane alone, demonstrating no improvement in outcome when using the combination (Figure 2A). Only 8 µM sulforaphane combined with vitamin D decreased the cell viability of PC-3 cells when compared with the compounds alone, with CI = 0.14 (Figure 2B).

### 3.2. Induction of Oxidative Stress

**Figure 3:**
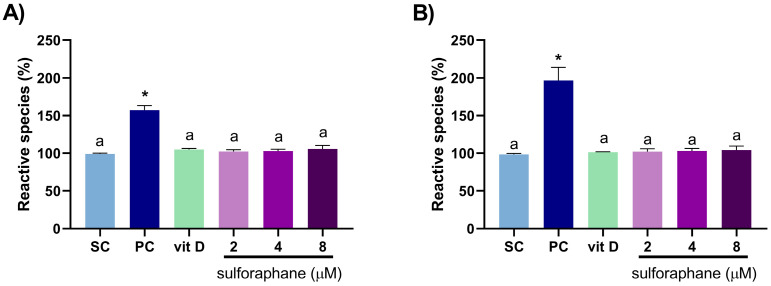
Analysis of reactive species production in DU145 (**A**) and PC-3 (**B**) cells after 3 h of treatment with sulforaphane or vitamin D, as assessed using the marker CM-H_2_DCFDA. Values that do not differ from each other share the same letter (one-way ANOVA followed by Tukey’s test, *p* < 0.05). * Different from the solvent control (Student’s *t*-test, *p* < 0.05). Vit D: 16 nM vitamin D; SC: solvent control (0.2% DMSO + 0.2% EtOH); PC: positive control (1 mM H_2_O_2_). Mean ± standard deviation (*n* = 3).

**Figure 4:**
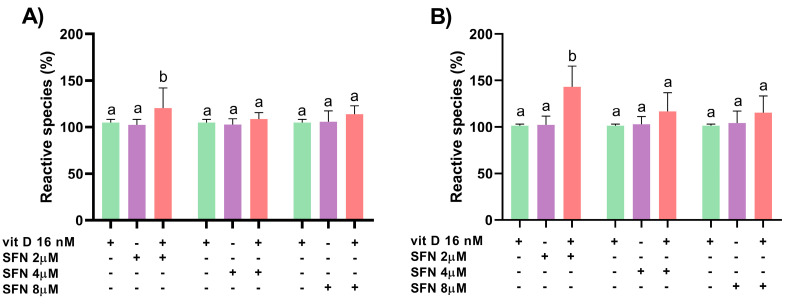
Analysis of reactive species production in DU145 (**A**) and PC-3 (**B**) cells after 3 h of treatment with sulforaphane (SFN) combined with vitamin D (vit D; 16 nM), as assessed using the marker CM-H_2_DCFDA. Data are expressed as mean ± standard deviation of three independent experiments. Distinct letters represent significant intra-group differences (two-way ANOVA followed by Tukey’s test, *p* < 0.05).

Vitamin D (16 nM) and sulforaphane (2, 4, and 8 µM) alone did not induce reactive species formation in DU145 and PC-3 cells after 3 h of treatment (Figure 3). The combination of 2 µM sulforaphane and vitamin D induced oxidative stress in DU145 (CI = 0.24) and PC-3 (CI = 0.05) cells (Figure 4).

### 3.3. Induction of Autophagy

**Figure 5:**
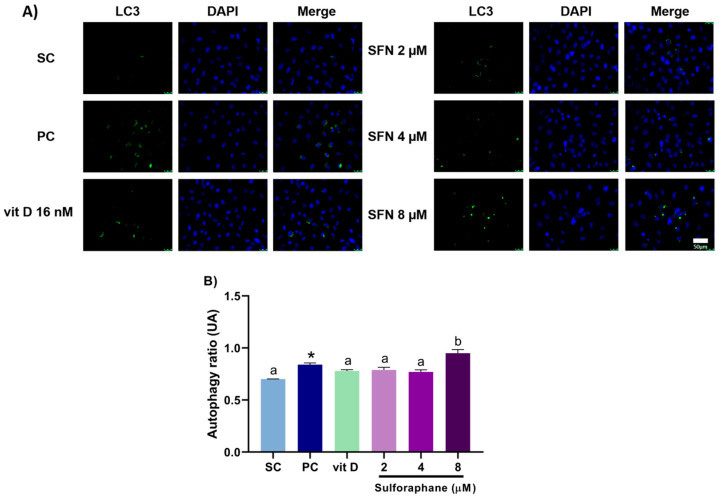
Autophagy rates in DU145 cells after 24 h of treatment with sulforaphane or vitamin D. (**A**) Photomicrographs show cell nuclei stained blue, and autophagosomes stained green. (**B**) Induction of autophagy in cells treated with sulforaphane (SFN; 2, 4, and 8 µM) or vitamin D (vit D; 16 nM) alone. SC: solvent control (0.2% DMSO + 0.2% EtOH), PC: positive control (100 μM chloroquine). Values that do not differ from each other share the same letter (one-way ANOVA followed by Tukey’s test, *p* < 0.05). * Different from the solvent control (Student’s *t*-test, *p* < 0.05). Mean ± standard deviation (*n* = 3). Scale bar = 50 µm.

**Figure 6:**
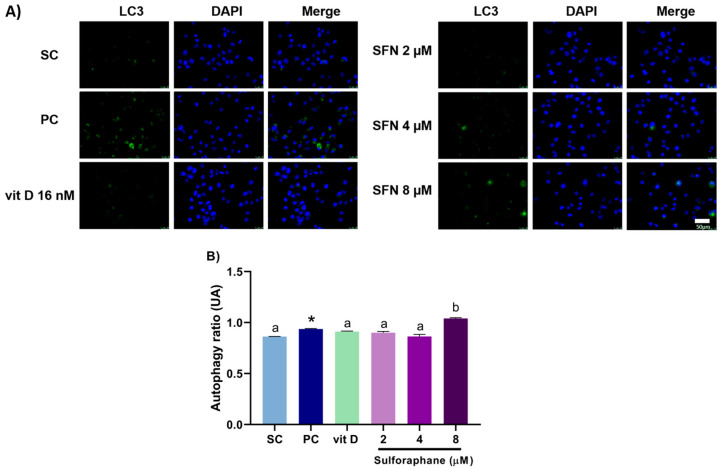
Autophagy rates in PC-3 cells after 24 h of treatment with sulforaphane or vitamin D. (**A**) Photomicrographs show cell nuclei stained blue, and autophagosomes stained green. (**B**) Induction of autophagy in cells treated with sulforaphane (SFN; 2, 4, and 8 µM) or vitamin D (vit D; 16 nM) alone. SC: solvent control (0.2% DMSO + 0.2% EtOH), PC: positive control (100 μM chloroquine). Values that do not differ from each other share the same letter (one-way ANOVA followed by Tukey’s test, *p* < 0.05). * Different from the solvent control (Student’s *t*-test, *p* < 0.05). Mean ± standard deviation (*n* = 3). Scale bar = 50 µm.

**Figure 7:**
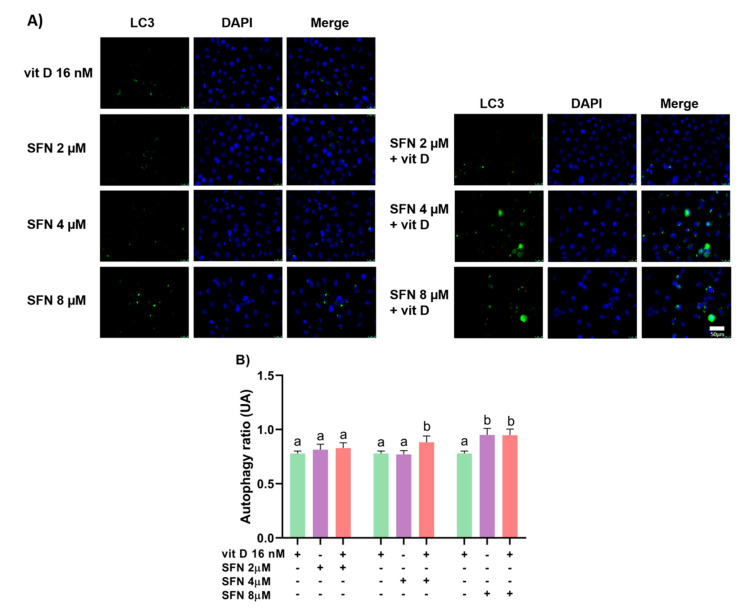
Autophagy rates in DU145 cells after 24 h of treatment with sulforaphane and vitamin D in combination. (**A**) Photomicrographs show cell nuclei stained blue, and autophagosomes stained green. (**B**) Induction of autophagy in cells treated with sulforaphane (SFN; 2, 4, and 8 µM) and vitamin D (vit D; 16 nM) in combination. Data are expressed as mean ± standard deviation of three independent experiments. Distinct letters represent significant intra-group differences (two-way ANOVA followed by Tukey’s test, *p* < 0.05). Scale bar = 50 µm.

**Figure 8:**
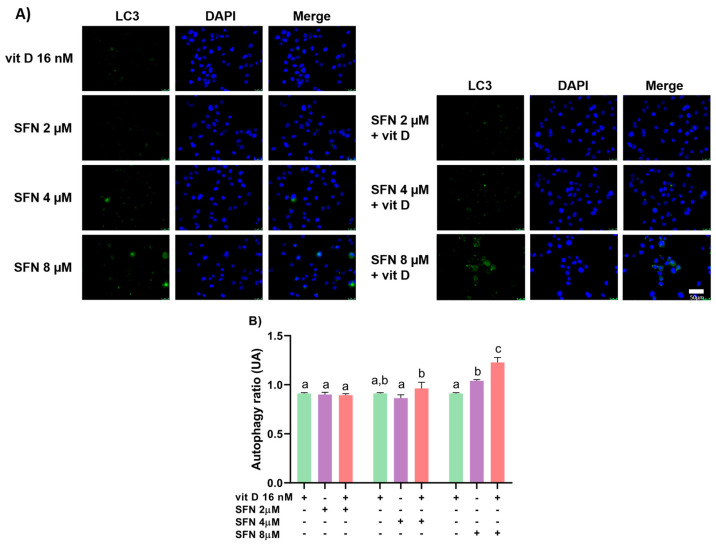
Autophagy rates in PC-3 cells after 24 h of treatment with sulforaphane and vitamin D in combination. (**A**) Photomicrographs show cell nuclei stained blue, and autophagosomes stained green. (**B**) Induction of autophagy in cells treated with sulforaphane (SFN; 2, 4, and 8 µM) and vitamin D (vit D; 16 nM) in combination. Data are expressed as mean ± standard deviation of three independent experiments. Distinct letters represent significant intra-group differences (two-way ANOVA followed by Tukey’s test, *p* < 0.05). Scale bar = 50 µm.

Sulforaphane alone at 8 µM enhanced the autophagy rate in DU145 (Figure 5) and PC-3 (Figure 6) cells after 24 h of treatment. In comparison with the compounds alone, sulforaphane at 4 µM combined with vitamin D (16 nM) increased the autophagy rate in DU145 cells, with CI = 0.88 (Figure 7), while sulforaphane at 8 µM combined with vitamin D (16 nM) augmented the autophagy rate in PC-3 cells, with CI = 0.85. The result of the combination of sulforaphane at 4 µM with vitamin D was the same as the treatment with vitamin D alone, not showing a better result when combined (Figure 8).

### 3.4. Induction of DNA Damage

**Figure 9:**
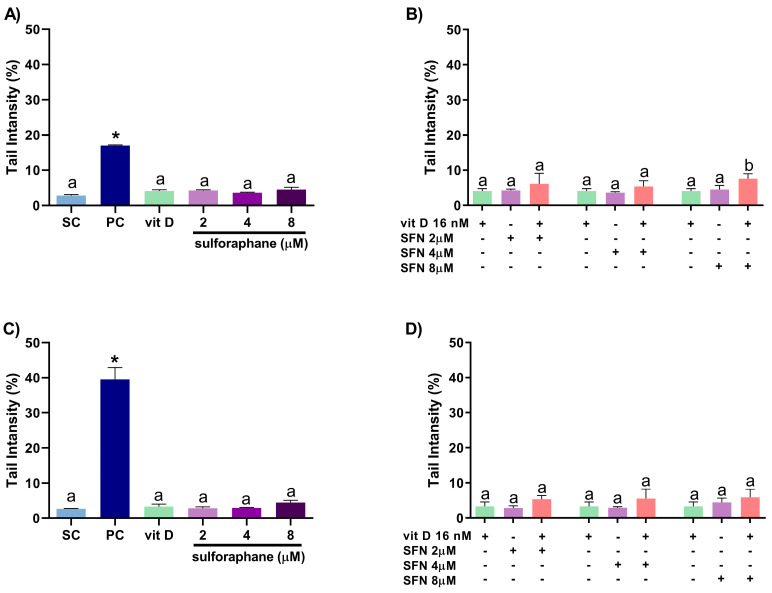
Percentage of DNA in the tail of DU145 (**A**) and PC-3 (**C**) nucleoids after treatment with vitamin D or sulforaphane for 24 h. Distinct letters represent significant differences (one-way ANOVA followed by Tukey’s test, *p* < 0.05). * Different from the solvent control (Student’s *t*-test, *p* < 0.05). Percentage of DNA in the tail of DU145 (**B**) and PC-3 (**D**) nucleoids after treatment with vitamin D combined with sulforaphane for 24 h. Distinct letters indicate significant intra-group differences (two-way ANOVA followed by Tukey’s test, *p* < 0.05). SC: solvent control (0.2% DMSO + 0.2% EtOH); PC: positive control (150 µM MMS); SFN: sulforaphane; vit D: 16 nM vitamin D. Mean ± standard deviation (*n* = 3).

Sulforaphane (2, 4, or 8 µM) and vitamin D (16 nM) alone did not induce DNA damage in DU145 (Figure 9A) and PC-3 (Figure 9C) cells, as assessed using the comet assay. Compared with the compounds alone, sulforaphane at 8 µM combined with vitamin D (16 nM) increased DNA damage in DU145 cells, with CI = 0.60 (Figure 9B). The sulforaphane–vitamin D combination did not induce significant DNA damage in PC-3 cells relative to the compounds alone (Figure 9D).

### 3.5. Analysis of Protein Expression

**Figure 10:**
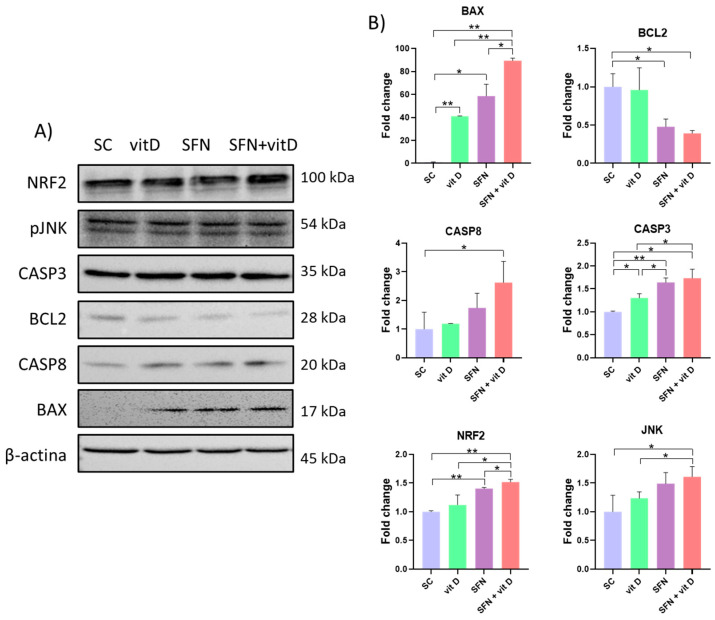
Protein expression in DU145 cells treated with sulforaphane and vitamin D for 24 h, as determined using Western blot. (**A**) Representation of protein bands analyzed using Western blot. (**B**) Fold-change of protein band quantification using ImageJ software and β-actin as endogenous control. * *p* < 0.05 and ** *p* < 0.01 (Student’s *t*-test). SC: solvent control (0.2% DMSO + 0.2% EtOH); SFN: sulforaphane (8 µM); vit D: vitamin D (16 nM).

Compared with the control group, the sulforaphane–vitamin D combinations upregulated expression of BAX, CASP8, CASP3, NRF2, and JNK, and downregulated expression of BCL2 in DU145 cells. Compared with the compounds alone, the sulforaphane–vitamin D combinations upregulated expression of BAX and NRF2 (Figure 10).

**Figure 11:**
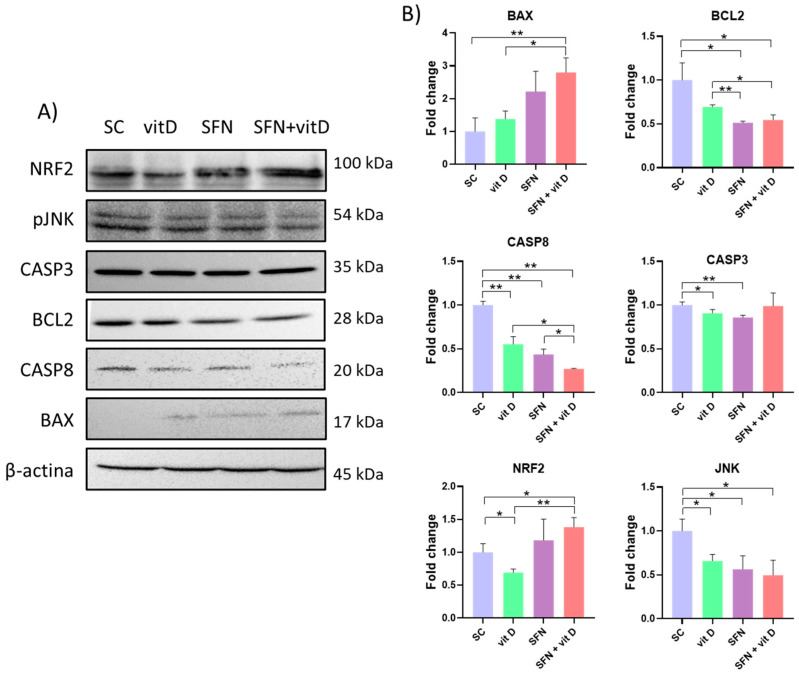
Protein expression in PC-3 cells treated with sulforaphane and vitamin D for 24 h, as determined using Western blot. (**A**) Representation of protein bands analyzed using Western blot. (**B**) Fold-change of protein band quantification using ImageJ software and β-actin as endogenous control. * *p* < 0.05 and ** *p* < 0.01 (Student’s *t*-test). SC: solvent control (0.2% DMSO + 0.2% EtOH); SFN: sulforaphane (8 µM); vit D: vitamin D (16 nM).

The sulforaphane–vitamin D combinations also upregulated expression of BAX and NRF2 and downregulated expression of BCL2, CASP8, and JNK in PC-3 cells, when compared with the control group. Compared with the compounds alone, the sulforaphane–vitamin D combinations significantly lowered CASP8 expression (Figure 11).

### 3.6. Analysis of Interactions between Compounds and Analyzed Proteins

**Figure 12:**
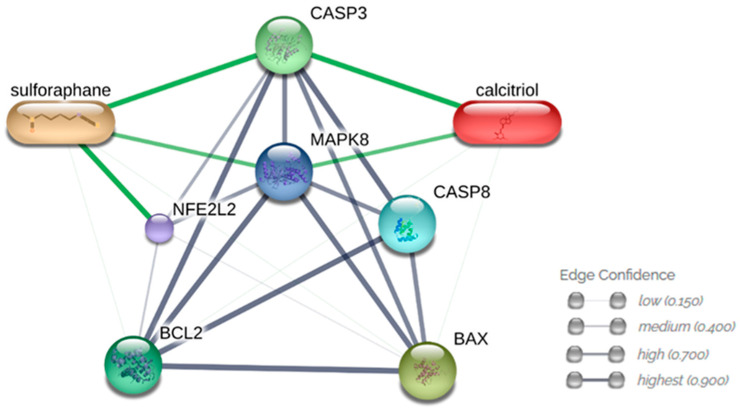
The STITCH database was used to predict interactions between sulforaphane, vitamin D, and proteins. Interactions between proteins are represented by gray lines, while interactions between compounds and proteins are represented by green lines. Source: STITCH (http://stitch.embl.de (accessed on 20 February 2023)).

The use of the STITCH database enabled prediction of direct interactions of sulforaphane and vitamin D (calcitriol) with CASP3 (0.947 and 0.953) and JNK (MAPK8—0.743 and 0.733). Sulforaphane directly interacts with NRF2 (NFE2L2—0.978). CASP8, BCL2 and BAX proteins interact with JNK (0.807, 0.999, and 0.961) and CASP3 (0.998, 0.990, and 0.869). No direct interaction between sulforaphane and vitamin D was detected (Figure 12).

## 4. Discussion

Patients with prostate cancer initially respond to therapy, but the tumor eventually progresses to more advanced aggressive forms [35]. Despite the successful results of current conventional therapies, there are still issues such as progression to metastasis, high toxicity associated with treatment, and development of drug resistance. These limitations have prompted the search for new non-toxic therapeutic strategies focused on the potential anticancer effect of nutraceuticals [7]. Considering that the combination of nutraceuticals can enhance anticancer effects [6], we hypothesized that sulforaphane and vitamin D in combination could have a stronger therapeutic effect than the compounds alone.

The tested concentrations of sulforaphane (2, 4, and 8 µM) and vitamin D (16 nM) did not alter the viability of DU145 and PC-3 cells after 24 h of treatment. Compared with the compounds alone, vitamin D and sulforaphane at 4 and 8 µM in combination decreased the viability of DU145 cells (Figure 2A), while only the combination of 8 µM sulforaphane and vitamin D decreased the viability of PC-3 cells (Figure 2B). Our findings corroborate literature reports of the decreased cell proliferation of DU145 and PC-3 cells treated with sulforaphane (1–20 µM) for 72 h, but not for 24 and 48 h [36], as well as the lack of effect of vitamin D (10 nM) on the cell proliferation of DU145 and PC-3 cells treated for 24 h [37].

Sulforaphane and its metabolites, sulforaphane-cysteine and sulforaphane-N-acetyl-cysteine, can trigger dynamic imbalance and microtubule disruption, leading to apoptosis of DU145 and PC-3 cells [38]. In hepatocarcinoma cells (HepG2), sulforaphane induces cell death via apoptosis, cell cycle arrest (G2/M phase), and DNA damage, inhibits clonogenic activity, and modulates the MAPK and AKT pathways [39]. Sulforaphane upregulates expression of proteins associated with endoplasmic reticulum stress, and induces apoptosis through activation of these signaling pathways and cell cycle modulation. This compound acts on key regulators such as cyclins, cyclin-dependent kinases (CDKs) and CDK inhibitors in a cell type-, dose-, and treatment time-dependent manner, inducing cell cycle arrest [10].

Treatment with sulforaphane combined with vitamin D upregulated expression of BAX and NRF2 proteins and downregulated BCL2 protein expression in both cell lines; increased expression of CASP8, CASP3, and JNK proteins in DU145 cells; and lowered expression of CASP8 and JNK proteins in PC-3 cells. The differences in target modulation may be due to cell line-specific mutations, such as TP53 and PTEN [40].

The combination of nutraceuticals modulated both the intrinsic (BAX/BCL2) and extrinsic (CASP8 and CASP3) apoptosis pathways in DU145 cells, and only the intrinsic pathway in PC-3 cells. Rutz et al. observed decreased cell viability, proliferation, clonogenicity, and cell cycle arrest, and increased histone acetylation in sulforaphane-treated DU145 and PC-3 cells; however, sulforaphane did not modulate intracellular signaling in the two cell lines through the same mechanisms. Apoptosis of PC-3 cells seemed to be independent of caspase activation, while apoptosis of DU145 cells was associated with increased expression of caspases 3 and 8 [36].

The p53 protein is an important tumor suppressor that modulates several functions such as DNA repair, cell cycle, and apoptosis. Mutations in the p53 gene occur at a high frequency in castration-resistant prostate cancer, suggesting p53 mutants may be possible targets for therapeutic interventions in the disease. PC-3 cells are p53-null, but DU145 cells co-express two different mutants, p53P223L and p53V274F. Phenethyl isothiocyanate, found in cruciferous vegetables, inhibits proliferation of DU145 cells selectively, and dependent on the type of mutation in p53, induces apoptosis and cell cycle arrest in the G1 phase, restores the p53P223L mutant from DU145 cells, and activates p53 targets such as BAX, p21, PUMA, and MDM2 [41]. The effect of combinations of nutraceuticals can also depend on the genotype of tumor cells. The synergistic effect of resveratrol and equol is PTEN-dependent in DU145 cells (PTEN+), compared with PC-3 (PTEN-) and DU145 cells (PTEN knockdown) [42]. Thus, it is important to test compounds in cell lines bearing different mutations. The p53 and PTEN mutations may explain some of the differences between outcomes in PC-3 and DU145 cells in the present study.

JNK (N-terminal c-Jun kinase) plays an important role in the growth of prostate carcinoma in vitro and in vivo, through the control of processes such as apoptosis, proliferation, migration, survival, differentiation, and inflammation via activation of several molecules. Many stimuli can activate JNK, including oxidative stress, toxins, and drugs. JNK activation triggers the caspase 8-mediated cascade to induce apoptosis, but JNK inhibition decreases cell proliferation and induces apoptosis in prostate cancer cells [43]. In this study, the sulforaphane–vitamin D combination activated JNK and CASP8 in DU145 cells, but inhibited these targets in PC-3 cells. Despite the differences in target modulation, both cell lines responded similarly to treatment.

Sulforaphane decreases the number of viable cells through activation of JNK-mediated signaling, generating ROS, causing cell cycle arrest and caspase-dependent apoptosis in DU145 cells [44]. Vitamin D seems to regulate the entire tumorigenesis process, from initiation to metastasis. Modulating cell-microenvironment interactions such as angiogenesis, antioxidant effects, and inflammation [45].

Regulation of reactive species levels is crucial for cellular maintenance. Moderate levels favor the control of cell proliferation and differentiation, while increased production or decreased clearance capacity result in oxidative stress, a condition that can cause extensive damage and cell death. Hence, an alternative anticancer strategy is the use of antioxidant enzyme inhibitors or agents capable of increasing the production of reactive species in order to cause tumor cell death [46].

Sulforaphane and vitamin D alone did not induce oxidative stress, but 2 µM of sulforaphane combined with vitamin D enhanced reactive species levels. The altered redox homeostasis of DU145 and PC-3 cells may be related to the induction of autophagy and DNA damage, as the accumulation of reactive species disturbs redox homeostasis and can damage cellular components, such as lipids, proteins, and DNA; severe damage to tumor cells results in cell death by apoptosis, autophagy, or necroptosis [47]. Sulforaphane can simultaneously activate autophagy and detoxification pathways, and trigger a cellular defense mechanism against oxidative stress via NRF2 activation [48]. Sulforaphane combined with vitamin D upregulated NRF2 expression in both cell lines. NRF2 activation may have participated in the autophagy induction in the present study.

Autophagy is an essential intracellular process that involves the engulfment of cellular components by autophagosomes, which subsequently fuse with lysosomes for degradation. It serves as a crucial cytoprotective mechanism for maintaining cellular homeostasis and recycling cytoplasmic contents. Recent evidence suggests that autophagy plays a pivotal role in cell death and has significant implications for various physiological processes in mammals, including tumor suppression. Compounds that induce the generation of ROS have been found to trigger autophagic cell death in tumor cells [49].

Treatment with sulforaphane increased autophagic activity in both prostate cancer cell lines. Sulforaphane induces autophagy in PC-3 and LNCaP prostate tumor cells [50]. Cytotoxic effects and oxidative stress-dependent induction of autophagy occur in pancreas cancer cells [51] and neuronal cells [52]. Vitamin D alone did not induce autophagy in DU145 and PC-3 cells, but it increased autophagy rates when combined with sulforaphane at 4 µM and 8 µM, respectively. The sulforaphane–vitamin D combination induces autophagy in the small intestine of mice [24]. Our findings suggested that vitamin D potentiated the effect of sulforaphane in inducing autophagy-associated cell death in DU145 and PC-3 cells.

Sulforaphane and its metabolites induce DNA damage in MG-63 osteosarcoma cells [53], HepG2 hepatocarcinoma cells [39], and MCF-7, MDA-MB-231, and SK-BR-3 breast cancer cells [54]. In contrast, 2, 4, and 8 µM sulforaphane alone did not induce DNA damage in both prostate tumor cell lines, but increased DNA damage in DU145 cells when combined with vitamin D. The augmented DNA damage probably results from induction of cell death, due to the elevated expression of the BAX, CASP3, and CASP8 proteins related to apoptotic cell death in DU145 cells.

Vitamin D combined with sulforaphane can modulate the WNT signaling pathway and inhibit histone deacetylases in Caco-2 colorectal cancer cells and TMX2–28 breast cancer cells [55,56]. This nutraceutical combination also induces autophagy in Apc1638N mice, which is heterozygous for germline mutation in the *Apc* gene and used to study intestinal tumorigenesis [24]. The findings of the present study with achievable plasma concentrations corroborate those described in the literature regarding the anticancer potential of sulforaphane and vitamin D in combination. The interaction prediction analyses indicated that the effects of sulforaphane and vitamin D in combination were related to modulation of JNK, one of the dysregulated MAPK signaling pathways in cancer. Proteins related to cell death regulation and oxidative stress were some of the JNK target proteins whose expression was altered.

Pharmacodynamic interactions can occur when compounds are administered together. They usually result from the combination of two compounds with similar mechanisms of action, and may result in reduced efficacy (antagonism), increased toxicity of one or both (synergism), or a previously unobserved effect that is not related to either of the two substances (coalism) [57]. When only one of the substances is active and the effect of the combination is greater, it is called potentiation [34]. In this study, coalism was detected in the induction of autophagy and DNA damage in DU145 cells, and potentiation was detected in the induction of autophagy in PC-3 cells. Coalism was detected in both cell lines in the cytotoxicity and production of reactive species.

In summary, sulforaphane combined with vitamin D increased cytotoxicity, oxidative stress, DNA damage, and autophagy through activation of the JNK pathway, upregulated expression of BAX, CASP8, CASP3 (pro-apoptotic), and NRF2 (antioxidant) proteins, and downregulated expression of BCL2 protein (anti-apoptotic) in DU145 cells. In PC-3 cells, the combination of nutraceuticals enhanced cytotoxicity, oxidative stress, and autophagy through upregulated expression of BAX and NRF2, and downregulated expression of JNK, CASP8, and BCL2. Modulation of the JNK/MAPK pathway seems to mainly mediate the effects of the combination of sulforaphane and vitamin D.

Although the results demonstrate the potential therapeutic benefits of combining sulforaphane and vitamin D, the observed effects are currently limited, highlighting the need for additional research to optimize their efficacy. Promising approaches are the utilization of nanocarriers and the development of analog compounds, which can improve the bioavailability, stability, and solubility of these compounds, thereby reducing adverse effects and enhancing the anticancer properties.

## 5. Conclusions

The combination of vitamin D with sulforaphane at human plasma concentrations is a potential alternative approach to the therapy of advanced prostate cancer because it promotes cytotoxicity, induces reactive species production, autophagy, and DNA damage, and modulates the JNK/MAPK pathway. These findings encourage further studies to explore and better understand the mechanisms of action of this combination.

## Figures and Tables

**Table 1:** List of antibodies used to analyze protein expression via Western blot.

Antibodies	Host	Supplier	CAT Number
Anti-β-Actin	Rabbit	Cell Signaling Technology, (Danvers, MA, USA)	8457S
Anti-Bax	Rabbit	Cell Signaling Technology	2772S
Anti-Bcl-2	Rabbit	Cell Signaling Technology	2876S
Anti-Caspase-3	Rabbit	ABclonal (Woburn, MA, USA)	A2156
Anti-Caspase-8	Rabbit	ABclonal	A0215
Anti-Nrf2	Rabbit	ABclonal	a1244
Anti-phospho-JNK1/2	Rabbit	ABclonal	ap0473
HRP Goat Anti-Rabbit IgG	Goat	ABclonal	AS014

## Data Availability

Not applicable.
